# Elongation factor 2 kinase promotes cell survival by inhibiting protein synthesis without inducing autophagy

**DOI:** 10.1016/j.cellsig.2016.01.005

**Published:** 2016-04

**Authors:** Claire E.J. Moore, Xuemin Wang, Jianling Xie, Jo Pickford, John Barron, Sergio Regufe da Mota, Matthias Versele, Christopher G. Proud

**Affiliations:** a Centre for Biological Sciences, University of Southampton, Southampton, SO17 1BJ, United Kingdom; b South Australian Health & Medical Research Institute, North Terrace, Adelaide, SA5000, Australia; c School of Biological Sciences, University of Adelaide, Adelaide SA5005, Australia; d Janssen Research & Development, Turnhoutseweg 30, 2340 Beerse, Belgium

**Keywords:** eEF2K, Translation, Glucose starvation

## Abstract

Eukaryotic elongation factor 2 kinase (eEF2K) inhibits the elongation stage of protein synthesis by phosphorylating its only known substrate, eEF2. eEF2K is tightly regulated by nutrient-sensitive signalling pathways. For example, it is inhibited by signalling through mammalian target of rapamycin complex 1 (mTORC1). It is therefore activated under conditions of nutrient deficiency.

Here we show that inhibiting eEF2K or knocking down its expression renders cancer cells sensitive to death under nutrient-starved conditions, and that this is rescued by compounds that block protein synthesis. This implies that eEF2K protects nutrient-deprived cells by inhibiting protein synthesis. Cells in which signalling through mTORC1 is highly active are very sensitive to nutrient withdrawal. Inhibiting mTORC1 protects them. Our data reveal that eEF2K makes a substantial contribution to the cytoprotective effect of mTORC1 inhibition.

eEF2K is also reported to promote another potentially cytoprotective process, autophagy. We have used several approaches to test whether inhibition or loss of eEF2K affects autophagy under a variety of conditions. We find no evidence that eEF2K is involved in the activation of autophagy in the cell types we have studied.

We conclude that eEF2K protects cancer cells against nutrient starvation by inhibiting protein synthesis rather than by activating autophagy.

## Highlights

• Eukaryotic elongation factor-2 kinase helps cancer cells survive nutrient starvation and is an attractive anti-cancer target.
• eEF2K has been reported to promote autophagy; however, our evidence shows eEF2K does not modulate autophagy.
• eEF2K protects cells by inhibiting protein synthesis.

## 1 Introduction

Recent work has identified eukaryotic elongation factor 2 kinase (eEF2K) as playing a key cytoprotective role in cancer cells under conditions of nutrient starvation [1], implying that inhibiting its activity may offer a novel therapeutic avenue in oncology. With this in mind, it is crucial to understand how eEF2K exerts this cytoprotective function.

eEF2K phosphorylates and inactivates eEF2, the protein required to help move the ribosome along the mRNA during translation elongation [2]. eEF2K belongs to a small group of atypical protein kinases, termed α-kinases [3]. To date, eEF2 is the only protein, other than eEF2K itself [4], [5], that is known to be phosphorylated by eEF2K. Protein synthesis consumes a large amount of energy [6], almost all (> 99%) of it in the elongation process. Elongation also uses almost all the amino acids consumed by protein synthesis. Consistent with this, the activity of eEF2K and the phosphorylation of eEF2 are increased under conditions of energy depletion or amino acid starvation [1], [2], [7].

Energy depletion (such as starvation of cells for glucose) can activate eEF2K via the AMP-activated protein kinase, AMPK [7], while amino acid starvation causes the inhibition of signalling through mammalian target of rapamycin complex 1 (mTORC1), a negative regulator of eEF2K. As noted, recent data show that eEF2K is important for the ability of cancer cells to withstand nutrient deprivation [1], and promotes breast cancer [8]. These recent discoveries may mean that eEF2K is a potential target for therapeutic intervention to tackle poorly-vascularised solid tumours. Since eEF2K slows down the elongation stage of mRNA translation, eEF2K may protect nutrient-deprived cells by decreasing the demands of protein synthesis for nutrients and energy.

Autophagy is a degradative process which can also protect cells during nutrient starvation and, like eEF2K, is negatively regulated by mTORC1 signalling [9]. Autophagy can break down macromolecules into their component building blocks and may therefore help cells to withstand periods of amino acid starvation. Activation of AMPK can also promote autophagy, likely by phosphorylating ULK1, an upstream regulator of autophagy ([10], [11], [12], [13]; see [14] for a review). Signalling through the mammalian target of rapamycin complex 1 (mTORC1) also regulates autophagy, in that case to restrain it [9]. Several studies have suggested that eEF2K may regulate (promote) autophagy in glioma cells [15], [16], [17] or mouse embryonic fibroblasts [18], such that it might provide a link between mTORC1 signalling and the regulation of autophagy. Dysregulation of autophagy is one of the hallmarks of cancer, although the role of autophagy in cancer is complex [19]. Autophagy may hinder tumorigenesis initially, but subsequently aid the survival of cancer cells in established tumours [19], [20]. However, no mechanism has so far been identified to explain how eEF2K could control autophagy.

The emerging data which show that eEF2K protects cancer cells against nutrient deprivation prompted us to examine how it exerts these cytoprotective effects. Our data provide strong evidence that eEF2K does so by inhibiting protein synthesis, and do not support the idea that eEF2K regulates autophagy, at least in the cell types we have studied. These findings are particularly important given the growing interest in eEF2K as a target for cancer therapy.

## 2 Experimental

### 2.1 Materials

Bradford assay reagent (500–-0001) was from Bio-Rad. Compounds JAN-384, -452, -613 and -849 were kindly provided Janssen Pharmaceutica NV (Beerse, Belgium). The properties of JAN-384 and the much less active analogue JAN-452 are described in [21]. JAN-613 is inactive against in vitro eEF2K at 30 μM; it was not tested against other kinases. JAN-849 was tested in a 234-kinase panel at Millipore, with following results (IC50): eEF2K, 16 nM; CLK2, 109 nM; GSK3α, 126 nM; GSK3β, 132 nM; CDK7, 269 nM; CDK9, 333 nM; all other kinases > 1000 nM. A patent covering these compounds was published in 2015 (WIPO Patent Application WO/2015/150557).

Signalling inhibitors and other compounds were obtained as follows: rapamycin (553211), Calbiochem; AZD8055 (S1555), MK2206 (S1078) and MG132 (S2619), Selleck; cycloheximide (CHX) (01810), Harringtonine (19079), chloroquine (C6628) and bafilomycin A1 (B1793), Sigma-Aldrich.

Primary antibodies: the P-eEF2 Thr56 antisera were custom made by Eurogentec (Belgium). Anti-eEF2K antibody was kindly provided by the Division of Signal Transduction Therapy, College of Life Sciences, University of Dundee, UK. Other antibodies were obtained as follows: eEF2 (catalogue number 2332), P-protein kinase B (PKB, also termed Akt, Ser473, 4060), P-ribosomal protein S6 240/244 (5364), LC3A/B (12741), poly ADP-ribose polymerase (PARP, 5625), all from Cell Signalling Technology; GAPDH (G8795), actin (A5441) and glutathione *S*-transferase (GST, GE27-4577-01) from Sigma-Aldrich.

### 2.2 Cell culture, transfection and treatment

Mouse embryonic fibroblasts (MEFs) from eEF2K^−/−^ (knockout) mice and matched wild-type counterparts were prepared from embryos at embryonic day 13.5. MEFs from eEF2K (WT) and eEF2K^−/−^ mouse embryos were cultured in Dulbecco's modified Eagle's medium (DMEM (41966); Invitrogen) supplemented with 10% foetal bovine serum (FBS, F2442, Sigma-Aldrich), 100 units/mL penicillin and 0.1 mg/mL streptomycin (15140–122, Invitrogen) at 37 °C in a humidified atmosphere of 5% CO_2_. Immortalised TSC2^−/−^ MEFs were generously provided by Dr. David Kwiatkowski (Harvard University, Boston). Human colon carcinoma HCT116 cell lines were cultured using standard procedures in a humidified incubator at 37 °C with 5% CO_2_ in McCoy's 5A media supplemented with 10% (v/v) FBS, 1.5 mM glutamine (25,030–081, Gibco), 100 units/mL penicillin and 0.1 mg/mL streptomycin. Human lung carcinoma A549 cell line containing an inducible shRNA plasmid directed towards the eEF2K mRNA was generously provided by Janssen Pharmaceutica NV and cells were cultured using standard procedures in a humidified incubator at 37 °C with 5% CO_2_ in DMEM media supplemented with 10% (v/v) FBS, 1.5 mM glutamine (25030-081, Gibco), 100 units/mL penicillin and 0.1 mg/mL streptomycin. To induce knockdown of eEF2K, cells were cultured for 5 days with 1 mM isopropyl β-D-1-thiogalactopyranoside (IPTG, I6758, Sigma-Aldrich) prior to experimentation.

After treatment, cells were lysed in ice-cold lysis buffer containing 1% (v/v) Triton X-100 (215680010, Acros Organics), 50 mM Tris (T6066-1KG, Sigma-Aldrich)–HCl (258148-2.5 L, Sigma-Aldrich) pH 7.4, 50 mM NaCl (BP358-212, Fisher Scientific), 0.2 mM EDTA (BP120-500, Fisher Scientific), 0.2 mM EGTA (E1102, Melford), 50 mM β-glycerophosphate (G9422, Sigma-Aldrich), 1 mM Na_3_VO_4_ (S6508, Sigma-Aldrich), 15 mM β-mercaptoethanol (125472500, Acros Organics) and 1 × protease inhibitor cocktail (5056489001, Roche). Lysates were spun at 16,000 ×* g* for 10 min at 4 °C; the supernatants were kept and total protein concentration was quantified by Bradford assay following the manufacturer's instructions.

### 2.3 SDS-PAGE and western blot analysis

These procedures were performed as described previously [22].

### 2.4 BHMT cleavage assay

A549 cells were transfected using lipofectamine LTX (15338100, Life Technologies) with a GST- betaine homocysteine methyltransferase (BHMT) reporter vector (kindly provided by Carol Mercer, University of Cincinnati, USA). 48 h after transfection cells were treated with AZD8055 (1 μM) for 16 h in the presence of E64d (6 μM, E8640, Sigma-Aldrich) and leupeptin (11 μM, L9783, Sigma-Aldrich). Cells were lysed as above. Total protein concentration was determined by Bradford assay and GST-BHMT was isolated using glutathione-sepharose (GE17-0756-01, Sigma-Aldrich). The precipitated GST-BHMT was washed three times in the ice cold lysis buffer. Precipitates were then boiled in SDS-PAGE sample buffer, resolved by SDS-PAGE and analysed by western blotting using anti-GST antibody.

### 2.5 Cell survival

Caspase 3/7 assays (G8090, Promega) were performed according to the manufacturer's instructions. Briefly, 10,000 cells/well were plated overnight in a 96-well plate. Cells were treated as described in the figure legends for the indicated time period. To measure caspase 3/7 activity, 50 μL of caspase Glo 3/7 reagent was added to each well for 2 h with constant shaking at room temperature. Luminescence was measured using a BMG Labtech FLUOstar Optimi plate reader. Cytotoxicity was evaluated by CellTox Green® cytotoxicity assay (Promega). Briefly, 10,000 cells/well were plated overnight in a 96-well plate. Cells were treated as described in the figure legends for the indicated time period. CellTox green dye was diluted 1/500 in test media and applied to cells for the times indicated in the figure. Fluorescence was measured at 485–500 nm_Ex_/520–530 nm_Em_ using a BMG Labtech FLUOstar Optimi plate reader.

### 2.6 Autophagic flux analysis

A549 cells were transfected with a vector encoding mCherry-EGFP-LC3B, a tandem fluorescent-tagged LC3 (a kind gift from Dr. Terje Johansen, Biochemistry Department, Institute of Medical Biology, University of Tromsø, Norway), using lipofectamine 3000 (L3000001, Life technologies) following manufacturer's instructions. After treatment cells were washed twice in phosphate buffered saline (PBS, 18912-014, Gibco) and then fixed in 4% formaldehyde (F8775, Sigma-Aldrich) for 15 min. Fixed cells were washed thrice in PBS and permeabilized with 0.1% Triton-X 100 for 5 min. Cells were then again washed thrice in PBS and mounted in ProLong® Gold Antifade Mountant (P36935, Life technologies). Cells were visualized using Leica TCS SP8X/MP microscope equipped with a tuneable white light laser using a 40 × oil immersion objective lens (NA = 1.30) numerical aperture objective. Green/red fluorescence ratio (GFP loss upon autolysosome formation) was measured using the Leica Application Suite X (LAS X) software (version 1.1.0).

### 2.7 Statistical analysis

All data were analysed by performing a two-way ANOVA with Tukey's multiple comparisons test for significance and, for the autophagic flux analysis, a two-way ANOVA with Dunnet's multiple comparisons test, using GraphPAD Prism 6 software. All experiments were performed at least three times with similar results.

## 3 Results

### 3.1 eEF2K is cytoprotective for cells faced with glucose starvation

**Fig. 1:**
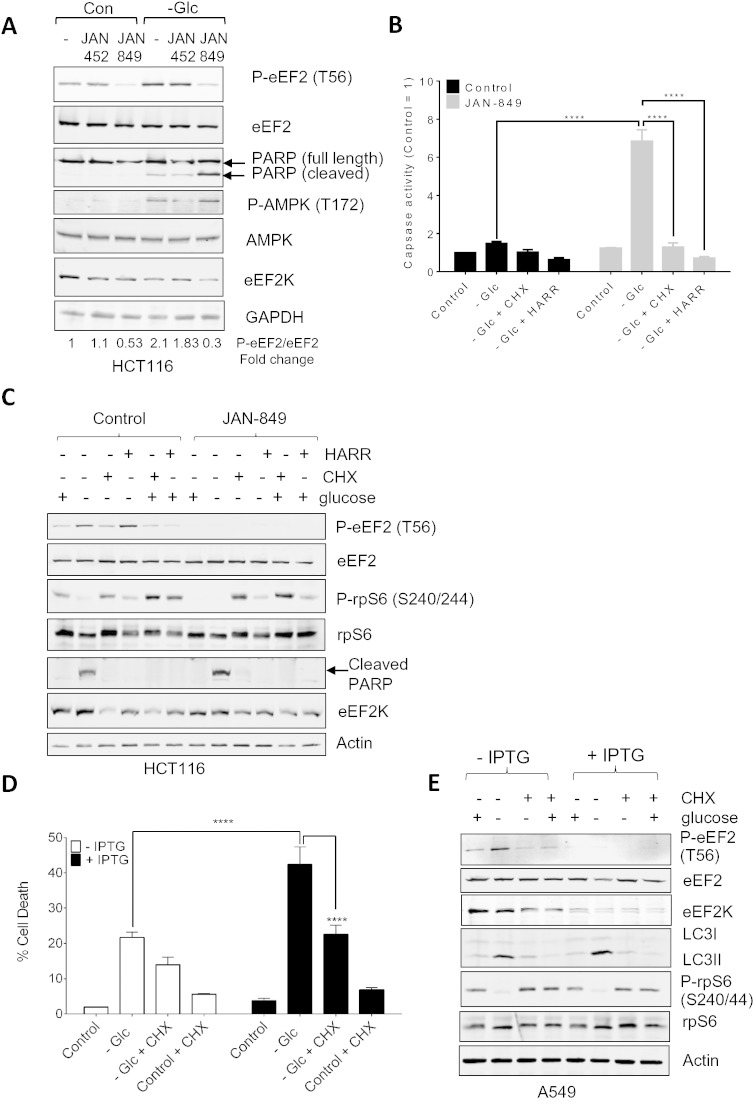
eEF2K helps cells withstand glucose starvation. (A) HCT116 cells were either kept in normal medium containing glucose or transferred to medium lacking glucose (− Glc) for 24 h in the absence or presence of JAN-452 or − 849 (3 μM). Cells were then lysed and samples containing equal amounts of protein were analysed by western blot using the indicated antibodies. Values given below each lane show quantitation of the signal for P-eEF2, corrected for the eEF2 signal from multiple experiments as in (A) expressed as P-eEF2 normalized to eEF2, mean ± SEM (control cells without treatment = 1; n = 3). (B) HCT116 cells were either kept in normal medium containing glucose or transferred to medium lacking glucose (− Glc) for 48 h in the absence or presence of JAN-384 and either cycloheximide (CHX; 5 μg/mL) or Harringtonine (HARR 2 μg/mL). Cells were lysed and caspase 3/7 activity was determined. Data are presented as means ± SEM (n = 3) ****, P < 0.001 for the indicated pairs of conditions. (C) As panel B, except cells were treated for 24 h and cell lysates were subjected to immunoblot analysis using the indicated antibodies. (D) A549 cells were cultured in the presence or absence of 1 mM IPTG for 5 days to induce the knockdown of eEF2K. A549 cells were either kept in normal medium containing glucose or transferred to medium lacking glucose (− Glc) for 48 h in the absence or presence of cycloheximide (5 μg/mL). IPTG was added to maintain eEF2K knockdown where indicated. Cell death was measured using CellTox Green® assay. Data are expressed relative to the control, mean ± SEM (control cells without treatment = 1; n = 3). (E) As (D), except cells were cultured for 24 h.

The compounds previously reported as inhibiting eEF2K are either highly non-selective (and can even increase eEF2 phosphorylation [23], [24]) or lack potency [24]. Janssen Pharmaceutica NV have recently developed a small set of highly specific and potent small-molecule inhibitors of eEF2K, together with closely-related but much less potent negative control compounds [25]. We made use of one such inhibitor (JAN-849) to explore the role of eEF2K in the ability of colorectal cancer (HCT116) cells to withstand deprivation for glucose, an important fuel for generating energy. As shown in Fig. 1A, starving HCT116 cells for glucose caused a modest, but consistent, increase in the phosphorylation of eEF2, indicative of activation of eEF2K. This may well be a consequence of the activation of the AMP-activated protein kinase, AMPK, which showed increased phosphorylation on its ‘T-loop’ (Thr172; Fig. 1A) in glucose-starved cells. AMPK is a positive regulator of eEF2K activity [7], [26] and generally acts to damp down energy-consuming processes in times of energy deficiency [27]. Under these conditions, glucose starvation only had a small effect on cell survival, as judged by the cleavage of poly ADP-ribose polymerase, PARP (Fig. 1A) and by caspase 3/7 activity (Fig. 1B). This clearly indicates that HCT116 cells possess a mechanism(s) to allow them to withstand glucose starvation.

We asked whether eEF2K played a role in this defence mechanism. Treating cells with the eEF2K inhibitor JAN-849 decreased eEF2 phosphorylation in glucose-starved cells (Fig. 1A), confirming the efficacy of JAN-849, while a less potent analogue, JAN-452, had almost no effect on eEF2 phosphorylation, consistent with its weak effect on eEF2K activity in vitro. Strikingly, treatment with the compound JAN-849 caused a marked enhancement of markers of cell death, i.e., PARP cleavage and the activity of caspases 3/7 (Fig. 1A, B) compared to cells starved of glucose in the absence of the inhibitor. JAN-849 only had a small effect on the survival of glucose-fed cells (Fig. 1B). These data strongly support the concept that eEF2K is cytoprotective in those nutrient-deprived cancer cells.

To assess whether the inhibition of eEF2K promotes cell death by disinhibiting translation elongation, we explored the effect of re-imposing inhibition of protein synthesis using two distinct translation inhibitors, cycloheximide (CHX, which inhibits elongation) and Harringtonine (Harr, which inhibits a late step in translation initiation [28], [29]). Each of these compounds restored cell survival in cells that had been starved of glucose and treated with the eEF2K inhibitor JAN-849 (Fig. 1B, C), presumably by inhibiting protein synthesis. It was important to assess whether these compounds were protecting cells by enhancing the phosphorylation of eEF2. As shown in Fig. 1C, Harringtonine had no effect on eEF2 phosphorylation and very little on mTORC1 signalling, while CHX actually caused a modest decrease in p-eEF2 again in line with its ability to activate mTORC1 signalling and enhance S6 phosphorylation [30], although other explanations are possible (see below). Taken together, these data provide strong evidence that JAN-849 does not promote cell death due to toxicity but rather by alleviating the inhibition of protein synthesis which is normally imposed by eEF2K in glucose-starved cells.

Thus, eEF2K protects cells against nutrient withdrawal by inhibiting protein synthesis. These data extend earlier observations where knocking down eEF2 was shown to help protect cells against nutrient starvation [1] by demonstrating directly that it is eEF2K's role in regulating the rate of protein synthesis that underlies its protective ability.

We considered it important to extend our study to other tumour-related cell lines. A line of A549 lung carcinoma cells harbouring an IPTG-inducible shRNA against the *EEF2K* mRNA was developed. The ability of A549 cells to withstand glucose starvation was decreased by knocking down eEF2K (Fig. 1D). To assess whether the role of eEF2K in aiding cell survival was due to inhibition of translation elongation, we tested the effect of CHX. If eEF2K protects cells by inhibiting elongation, adding CHX to cells in which eEF2K had been knocked down should help cell survival. As shown in Fig. 1D, CHX partially rescued the death induced by glucose starvation, suggesting that eEF2K does aid cell survival by inhibiting translation elongation. CHX caused a decrease in eEF2 phosphorylation again (Fig. 1E), consistent with its ability to activate mTORC1 signalling [30]. The fact that CHX treatment also decreased the levels of eEF2K (Fig. 1E) likely also contributes to the fall in eEF2 phosphorylation (Fig. 1E). The partial nature of the ‘rescue’ by CHX likely reflects the fact that CHX had an adverse effect on cell survival under control conditions (Fig. 1E), so that the overall level of cell survival is a ‘trade-off’ between the pro-survival and pro-death effects of this drug. Starvation for glucose induced the modification of LC3 but this was not impaired in cells where eEF2K had been knocked down (Fig. 1E).

These data indicate that eEF2K promotes cell survival by inhibiting protein synthesis, consistent with it acting through its only known substrate, eEF2. However, several studies using other cancer cell lines have reported that eEF2K promotes autophagy and thereby aids cell survival during nutrient starvation [15], [16], [17], [18].

### 3.2 eEF2K does not regulate autophagy in lung carcinoma cells

**Fig. 2:**
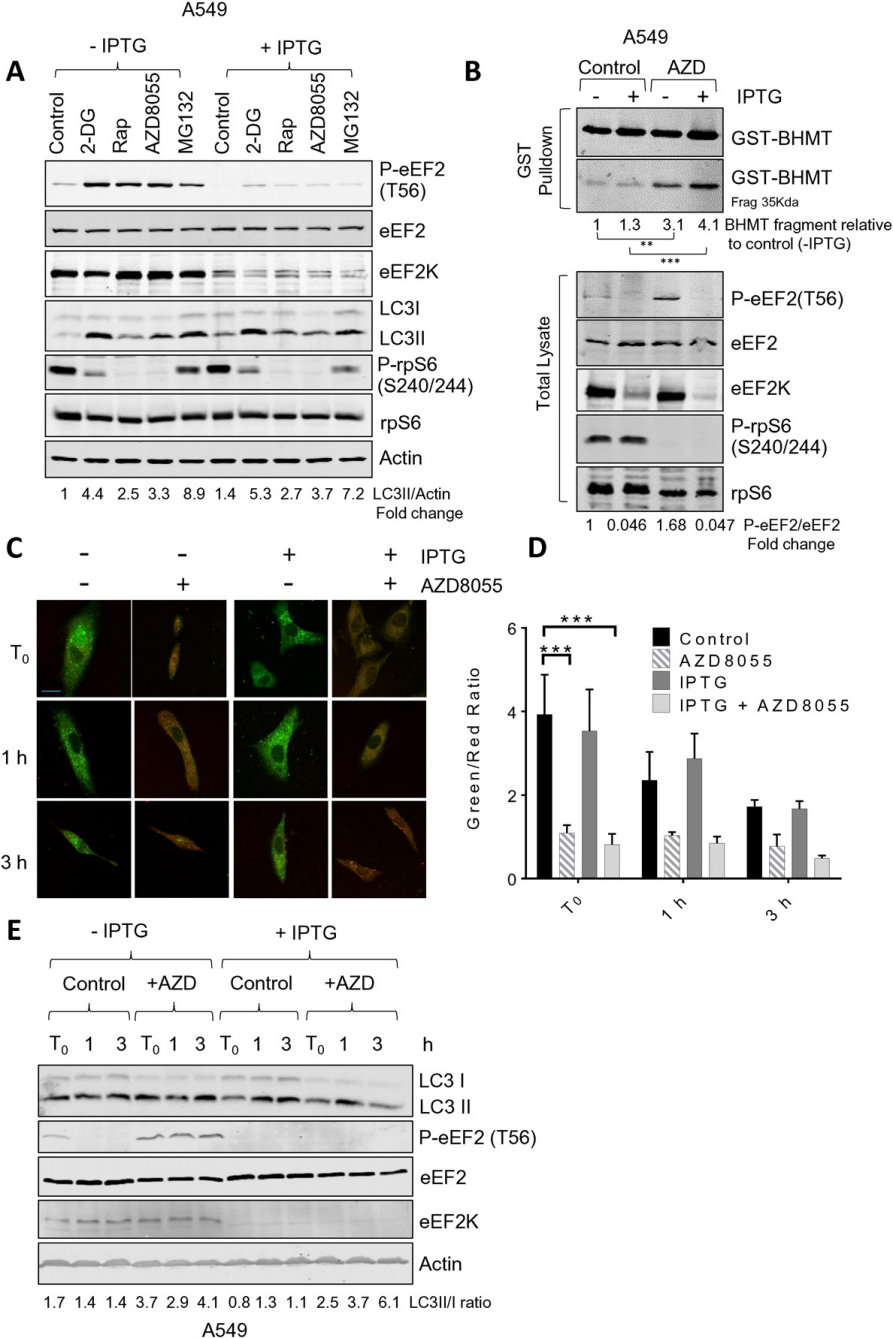
eEF2K does not regulate autophagy in lung carcinoma cells (A) A549 cells were cultured in the presence or absence of 1 mM IPTG for 5 days to induce the knockdown of eEF2K. Cells were then treated with 10 mM 2-deoxyglucose (2DG), rapamycin (100 nM), AZD8055 (1 μM) or MG132 (10 μM) for 16 h. Cells were then lysed and samples containing equal amounts of protein were analysed by western blot using the indicated antibodies. Values given below each lane show quantitation of the signal for LC3II, corrected for the actin signal from multiple experiments as in (A) expressed as LC3II normalized to actin, mean ± SEM (control cells without treatment = 1; n = 3). (B) A549 cells were cultured as in (A) to induce eEF2K knockdown. A549 cells were transiently transfected with the GST-BHMT construct and maintained in full media in the presence or absence of AZD8055 (1 μM) for 16 h, in the presence of leupeptin and E64d. GST-BHMT was precipitated with glutathione agarose from the whole cell extracts (bottom panel). Values given below show the quantitation of data from multiple experiments as in (B) expressed as the level of BHMT fragment normalized to the control without IPTG, mean ± SEM (control cells without treatment = 1; n = 3). Values given below each lane show quantitation of the signal for P-eEF2, corrected for the eEF2 signal from multiple experiments as in (B) expressed as P-eEF2 normalized to eEF2, mean ± SEM (control cells without treatment = 1; n = 3). (C) Cells were treated as described in Supplemental Fig. S3, visualized by confocal microscopy and the rate of autophagosome–lysosome fusion was measured according to green/red fluorescence ratio which reflect the percentage of unfused LC3. Scale bar = 20 μm. (D) Data quantification of C, data are presented as means ± SEM. (*n* = 5) ***P < 0.001, obtained by two-way ANOVA (Dunnett's test). (E) Untransfected A549 cells were treated with AZD8055 and BafA1 as described in A, cell lysates were collected at T_0_, 1 and 3 h for immunoblotting analysis. Values given below each lane show quantitation of the signal for LC3II, corrected for the LC3I signal from multiple experiments as in (E) expressed as LC3II normalized to LC3I, mean ± SEM (control cells without treatment = 1; n = 3).

In A549 cells, 2-DG, rapamycin, the mTOR kinase inhibitor AZD8055 [31] and the proteasome inhibitor MG132 all induced the phosphorylation of eEF2, indicative of activation of eEF2K (Fig. 2A). As shown, IPTG induced reductions in eEF2 phosphorylation and eEF2K levels in A549 cells (Fig. 2A). However, the ability of each of these treatments to induce the modification of LC3 was not significantly diminished by knocking down eEF2K expression (Fig. 2A); in fact, a slight increase was sometimes observed. (LC3 is modified by addition of ethanolamine as a key step in autophagosome formation [32], which causes it to migrate faster on SDS-PAGE indicating stimulation of this step in autophagy).

In the presence of chloroquine (CQ), to block autophagy downstream of LC3 modification, we also observed no difference in the extent of LC3 modification between A549 cells where eEF2K had been knocked down and corresponding control cells (Supplementary Fig. S1A, B); CQ increased modified LC3 to similar extents in all cases.

To further examine the role of eEF2K in regulating autophagy, A549 cells were treated with the eEF2K inhibitor (JAN-384). Inhibiting eEF2K activity using JAN-384 increased the levels of LC3II in response to mTOR inhibition using AZD8055 and rapamycin (Supplementary Fig. S2A, C. Interestingly autophagy induction by 2-DG was reduced in eEF2K inhibitor-treated cells although this was not significant.

Taken together, these data indicate that eEF2K plays no role in linking (inhibition of) mTORC1 signalling to the control of autophagy, as assessed by the levels of modified LC3.

### 3.3 eEF2K does not regulate autophagic flux

A number of reservations have been expressed about reliance on the modification of LC3 as a read-out for regulation of autophagy; for example, its modification is an intermediate step in the pathway, not an endpoint [33]. We therefore used an alternative assay looking for a product of autophagy, i.e., the cleavage of BHMT. BHMT is a substrate for the autophagic pathway and an assay for this has been developed using glutathione *S*-transferase (GST)-tagged BHMT [34]. The degree of cleavage of GST-BHMT gives an end-point readout of autophagic flux. Treating A549 cells with AZD8055 caused a marked increase in the phosphorylation of eEF2 and in cleavage of GST-BHMT (Fig. 2B). IPTG-induced knock down of eEF2K entirely eliminated the phosphorylation of eEF2 but did not prevent the cleavage of GST-BHMT; if anything, cleavage tended to be greater in the eEF2K knock-down cells. Cleavage of GST-BHMT was completely blocked by chloroquine indicating it is entirely due to autophagy, and no other proteolytic pathways contribute to this (data not shown). Thus, eEF2K is not required for the process of autophagy or its activation, at least downstream of mTOR.

We also examined the progression of autolysosome formation using a tandem fluorescently-tagged LC3 (mCherry-EGFP-LC3B) which allowed us to determine autophagosome and lysosome fusion by evaluating loss of GFP fluorescence. Green (GFP) but not red (mCherry) fluorescence is quenched by protonation in response to acidified intra-lysosomal pH upon autolysosome fusion [27]. 33 h after mCherry-EGFP-LC3B transfection, either vehicle control or the mTOR inhibitor AZD8055 was added to A549 cells for 16 h to evoke eEF2K activation as well as inducing autophagy (Fig. 2C), at the end of the 16 h incubation period (Supplemental Fig. S3), bafilomycin A1 (BafA1) was also added to the cells for 4 h to inhibit the fusion between autophagosomes and lysosomes and hence preventing the maturation of autophagic vacuoles. Upon releasing from the blockage of autolysome production in response to BafA1, there was a gradual decrease in green/red ratio within the next 3 h in vehicle treated A549 cells, indicative of the re-formation of autolysosomes, whereas in AZD8055 treated cells, green/red ratio remained low in comparison to the control cells, indicating an induction of autophagy and hence increased autolysosome formation (Fig. 2C, D). Although LC3II/I ratio basal levels were high in these cells, AZD8055 was still capable to further increase the LC3II/I ratio (Fig. 2E), suggesting an induction of autophagy. Nevertheless, the increase in autolysosome formation as a result of AZD8055 treatment of A549 cells was not affected by knocking down eEF2K upon the induction of shRNA in response to IPTG treatment (Fig. 2C, D), suggesting that eEF2K does not play role in the formation of autolysosomes under these conditions.

### 3.4 Activation of autophagy by different stimuli is not impaired in eEF2K-knock-out MEFs

**Fig. 3:**
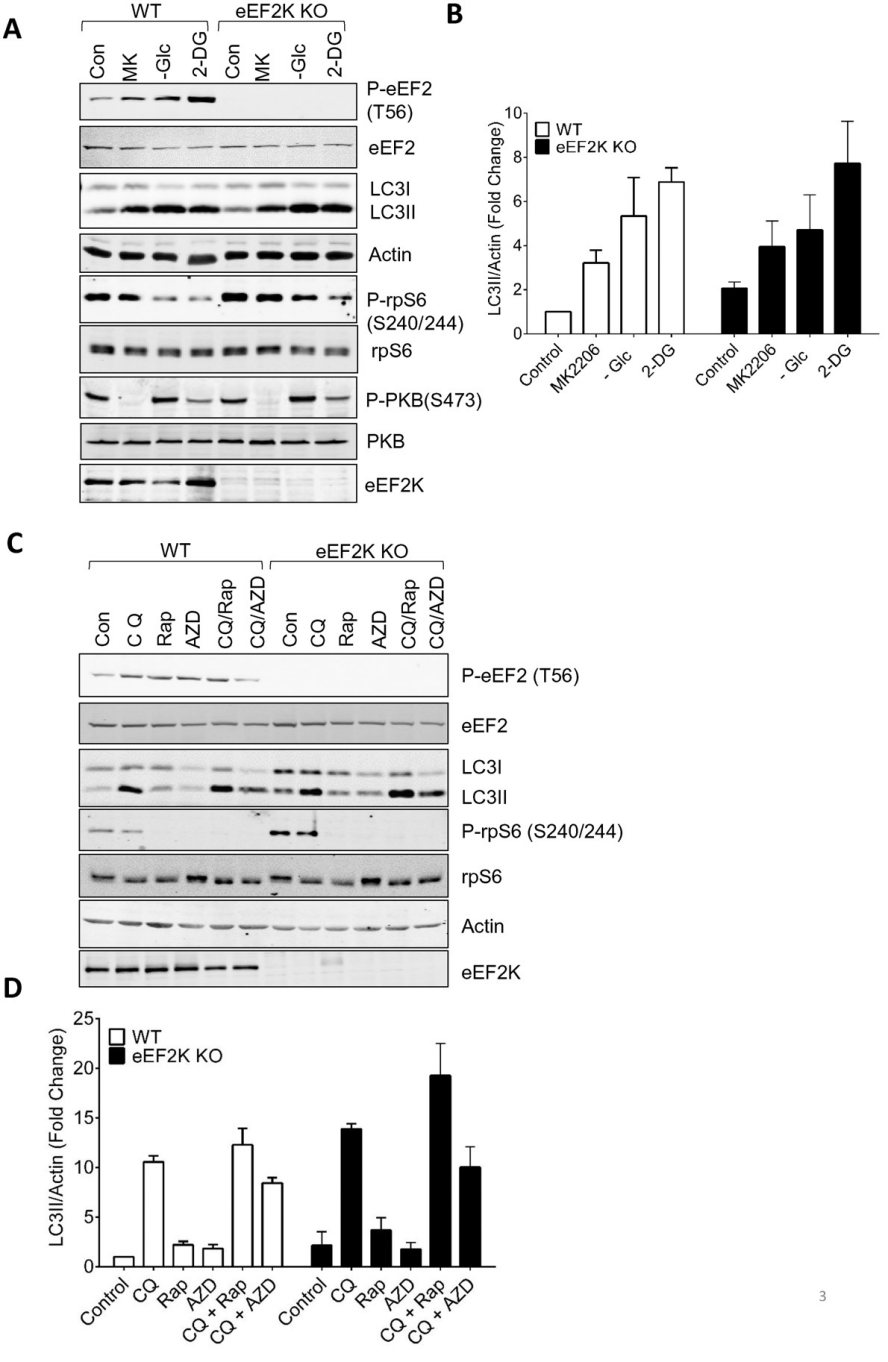
Role of eEF2K in autophagy in mouse embryonic fibroblasts (MEFs). Wildtype (WT) and eEF2K^−/−^ (KO) MEFs were treated, where indicated, with MK2206 (MK) (5 μM) or 10 mM 2-deoxyglucose (2DG) or in medium lacking glucose (− Glc) for 24 h. Cells were then lysed and samples containing equal amounts of protein were analysed by western blot using the indicated antibodies. (B) Quantitation of data from multiple experiments as in (A) expressed as LC3II normalized to actin, mean ± SEM (control cells without treatment = 1; n = 3). (C) Wild-type (WT) and eEF2K^−/−^ (KO) MEFs were treated, where indicated, with rapamycin (50 nM) or AZD8055 (0.5 μM) and/or chloroquine (10 μM) for 24 h. Cells were then lysed and samples containing equal amounts of protein were analysed by western blot using the indicated antibodies. (D) Quantitation of data from multiple experiments as in (C) expressed as LC3II normalized to actin, mean ± SEM (control cells without treatment = 1; n = 3).

In view of earlier findings, largely from glioma cells, we considered it important to assess whether eEF2K played a role in regulating autophagy in other cell types and situations. We exploited the availability of cells (embryonic fibroblasts) from mice in which the eEF2K gene has been disrupted (termed eEF2K^−/−^ (KO) MEFs). As expected, both the expression of eEF2K and the phosphorylation of eEF2 are lost in MEFs from eEF2K^−/−^ mice (Fig. 3A). Wild-type (WT) or eEF2K^−/−^ MEFs were treated in ways previously shown to activate autophagy, i.e., addition of the PKB/Akt inhibitor MK2206 [35], starvation for glucose or exposure to 2-deoxyglucose (2-DG), a metabolic ‘poison’ which causes depletion of ATP levels. Each of these treatments increased the level of phosphorylation of eEF2 in WT MEFs, with 2-DG having the greatest effect (Fig. 3A). The various treatments of WT MEFs caused a shift in the ratio of the two forms of LC3 from the upper (LC3I) to the lower band (LC3II, Fig. 3A). The effects of these conditions on LC3 were at least as marked in eEF2K^−/−^ MEFs as in WT cells (Fig. 3A; data are quantified for replicate experiments in Fig. 3B). These data indicate that eEF2K function is not required for the induction of autophagy under any of these conditions, in line with earlier findings for nutrient-deprived MEFs [1].

Since it is well established that mTORC1 signalling negatively regulates both autophagy and eEF2K, we studied whether eEF2K is involved in the control of autophagy by mTORC1. LC3-II is an intermediate in the process of autophagy, not an endpoint. Thus, if eEF2K regulated autophagy at multiple steps, LC3-II might well not provide a good read-out for the level of autophagic flux. We therefore used chloroquine (CQ), which inhibits autophagy downstream of LC3 (and its lipidation). As expected, CQ increased the levels of modified LC3-II (as it prevents its destruction during autophagy; Fig. 3C) in MEFs treated with rapamycin (Rap) or AZD8055 (AZD). Again, the absence of eEF2K did not prevent the modification of LC3; if anything, modification was slightly greater in eEF2K^−/−^ cells treated with rapamycin and CQ than in the corresponding controls (Fig. 3C, D). Thus, if anything, eEF2K acts to restrict LC3 modification in response to inhibition of mTORC1. Consistent with this, the basal level of LC3 modification was greater in eEF2K^−/−^ cells than in their wild-type counterparts [1]. Interestingly we also observe enhanced phosphorylation of S6; this might be due to increased autophagic production of amino acids which activate mTORC1 signalling [36], although other explanations are possible.

### 3.5 eEF2K contributes to the cytoprotective effect of rapamycin in cells with hyperactive mTORC1 signalling

Choo et al. [37] previously reported that cells lacking TSC2, a negative regulator of mTORC1 signalling, are particularly sensitive to glucose starvation and that rapamycin was cytoprotective under such conditions, acting by slowing down (energy-requiring) anabolic processes. Since eEF2K controls a major energy-consuming anabolic process (protein synthesis), is negatively regulated by mTORC1, and is thus stimulated by treatment of cells with rapamycin [38], we asked whether eEF2K played a role in these effects.

**Fig. 4:**
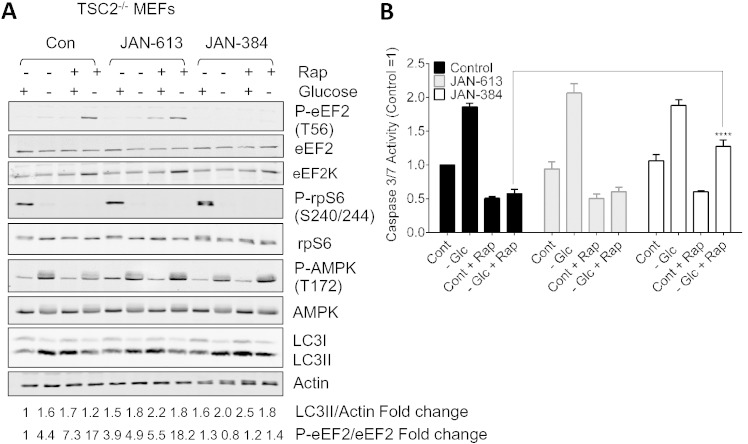
eEF2K aids the survival of TSC2^−/−^ cells under glucose-starved conditions. (A) TSC2^−/−^ MEFs were maintained in medium containing (control) or lacking glucose (− Glc) for 24 h in the presence of rapamycin (20 nM) and compounds JAN-613 (less active) or JAN-384 (highly active) (each at 3 μM). Cells were then lysed and samples containing equal amounts of protein were analysed by western blot using the indicated antibodies. Values given below each lane show quantitation of the signal for LC3II, corrected for the actin signal or P-eEF2, corrected for the eEF2 signal from multiple experiments as in (A), mean ± SEM (control cells without treatment = 1; n = 3). (B) Cells were treated for 48 h as described in (A), except that they were processed for caspase 3/7 assay.

As expected, eEF2 phosphorylation was low in TSC2^−/−^ cells (Fig. 4A). When these cells were starved of glucose, eEF2 phosphorylation remained low, presumably due to the high level of mTORC1 signalling. We previously reported that in TSC2^−/−^ cells that mTORC1 activation blocked the transcription of eEF2K [39]. The addition of rapamycin reverses this effect as a result the total levels of eEF2K protein increase. Therefore, the addition of rapamycin to cells starved of glucose caused a large increase in eEF2 phosphorylation (Fig. 4A). A more recently-developed, highly-active eEF2K inhibitor from Janssen Pharmaceutica NV, JAN-384, almost completely prevented this increase in eEF2 phosphorylation, while a closely-related less active analogue, JAN-613, did not.

To assess cell viability, we measured caspase 3/7 activity (Fig. 4B). Judged by both criteria, glucose starvation adversely affected the TSC2^−/−^ cells, and this effect was fully (Fig. 4B) countered by rapamycin, although in our hands the effects were consistently smaller in magnitude than those reported by Choo et al. [37], who starved their cells for longer (60 h). In the case of caspase activity, rapamycin decreased this in control conditions too. Importantly, the protective effect of rapamycin was lost when cells were treated with the highly active eEF2K inhibitor JAN-384, while JAN-613 had no effect (Fig. 4B).

The differing effects of JAN-613 and JAN-384 on both eEF2 phosphorylation and cell viability markers indicates that much, perhaps most, of the protective effect of rapamycin is mediated through eEF2K and thus presumably via inhibition of protein synthesis. The eEF2K-mediated inhibition of protein synthesis likely plays a major role in the ability of rapamycin to bring energy demand more closely into line with energy supply under glucose-starved conditions [37]. Several earlier studies have suggested that eEF2K positively regulates autophagy in other cell types [16], [17], [18], [40] where mTORC1 signalling is not hyperactivated. This effect might potentially contribute to the cytoprotective effects of rapamycin in glucose-starved TSC2^−/−^ cells. However, Choo et al. [37], using similar cells and protocols as those we have used, excluded a requirement for autophagy in the cytoprotection afforded by rapamycin. Glucose starvation did increase the modification of LC3 in TSC2^−/−^ MEFs, but this was not significantly affected by inhibiting eEF2K (Fig. 4A). Thus, it appears that the cytoprotective effect of eEF2K in this setting is not linked to regulation of autophagy.

## 4 Discussion

The present data add further support to the concept that eEF2K plays an important role in enabling cancer cells to survive glucose starvation, in particular by extending earlier work to show that (i) pharmacological inhibition of eEF2K, like knocking down its expression, impairs the ability of cells to withstand glucose starvation, (ii) this role extends to cancer cell types other than those previously tested (which were mainly glioma, see e.g., [1]) and (iii) that restoring inhibition of elongation in cells where eEF2K has been inhibited ‘rescues’ them from cell death, indicating that eEF2K protects cells by inhibiting protein synthesis. This is consistent with eEF2Ks ability to phosphorylate and inhibit its only known substrate, eEF2.

mTORC1 signalling, a negative regulator of eEF2K, is activated in a high proportion of cancers, leading to the idea that inhibiting mTORC1, using rapamycin or related compounds, may be an effective anti-cancer therapy. However, Choo et al. [37] showed that rapamycin actually promoted the survival of cells in which mTORC1 is hyper-activated (TSC2-null cells) when they are starved of glucose. Rapamycin promotes activation of eEF2K by blocking mTORC1 signalling. Here we show that inhibiting eEF2K substantially reverses the pro-survival effect of rapamycin in TSC2-null cells. This implies that eEF2K makes an important contribution to the pro-survival effects of rapamycin in this setting and that inhibition of eEF2K may be particularly effective in inducing the death of cells with hyperactive mTORC1 under conditions of nutrient shortage. These data lend further support to the concept that inhibiting eEF2K activity is likely useful in tackling solid tumours [1], [41].

Previous studies, many of them in glioma cells, have indicated that eEF2K is a positive regulator of autophagy, a process which can aid the survival of nutrient-starved cells [20], [42]. Given that mTORC1 signalling negatively regulates both eEF2K and autophagy, these data were of considerable interest; for example, they suggested that eEF2K might provide a link between mTORC1 and the control of autophagy. However, in the cell lines we have studied, we find no evidence that eEF2K positively regulates autophagy. In fact, in some cases there was a trend for the extent of LC3 modification, a key step in autophagy, to be greater in settings where eEF2K has been inhibited or knocked down, although the differences were modest. Consistent with our findings, a very recent study [43] reported that silencing of eEF2K actually promoted autophagy, at least under basal conditions, suggesting a negative link between eEF2K and autophagy in this situation.

Several studies have shown that mTORC1 can regulate ULK1 and other components that regulate autophagy (reviewed, [9]). It seems likely that these signalling links, rather than eEF2K, couple nutrient starvation and inhibited mTORC1 signalling to the induction of autophagy. In addition, other signalling links, e.g., via AMPK, can also couple nutrient deprivation to activation of autophagy [14].

Our data show that inhibiting protein synthesis rescues the impairment in cell survival seen in nutrient-deprived cells when eEF2K is inhibited. eEF2K has only one known substrate, eEF2, which is required for the energy-intensive process of translation elongation. Thus, the logical explanation for these observations is that activation of eEF2K contributes to cell survival by phosphorylating (and inhibiting) eEF2, thereby decreasing the demands of protein synthesis for energy and thus metabolic fuels such as glucose. These data are important for understanding the role of eEF2K in cancer cell biology and its relevance for cancer therapy.

## Appendix A Supplementary data

Fig. S1 Role of eEF2K in autophagy in A549 cells (A) A549 cells were cultured in the presence or absence of 1 mM IPTG for 5 days to induce the knockdown of eEF2K. Cells were treated, where indicated, with rapamycin (50 nM) or AZD8055 (0.5 μM) or chloroquine (10 μM) for 16 h. Cells were then lysed and samples containing equal amounts of protein were analysed by western blot using the indicated antibodies. (B) Quantitation of data from multiple experiments as in (C) expressed as LC3II normalized to actin, mean ± SEM (control cells without treatment = 1; n = 3). Fig. S2 Role of eEF2K in autophagy in A549 cells (A) A549 cells were cultured in the presence or absence of 3 μM JAN-384 for 30 minutes prior to treatment with 10 mM 2-deoxyglucose (2DG), rapamycin (100 nM), AZD8055 (1 μM) or MG132 (10 μM) for 16 h. Cells were then lysed and samples containing equal amounts of protein were analysed by western blot using the indicated antibodies. (B) Quantitation of data from multiple experiments as in (A) expressed as P-eEF2 normalized to eEF2, mean ± SEM (control cells without treatment = 1; n = 3). Statistical analysis refers to differences between individual treatments with/without JAN-384, *P < 0.05, **P < 0.01, ****P < 0.0001. (C) Quantitation of data from multiple experiments as in (A) expressed as LC3II normalized to actin, mean ± SEM (control cells without treatment = 1; n = 3). Fig. S3 eEF2K does not play a role in regulating autophagolysosome formation. Schematic representation of the experimental design in Fig. 2C. A549 cells (where indicated, in medium containing 1 mM IPTG to induce the expression of shRNA against eEF2K) were transfected with mCherry-EGFP-LC3B, a tandem fluorescently-tagged LC3. 33 h later cells were treated with vehicle (DMSO) or AZD8055 (1 μM) for 16 h, before the addition of Bafilomycin A1 (BafA1, 50 nM). BafA1 were removed 4 h post-treatment while the vehicle/AZD8055 and IPTG were re-added to the cells. Samples were collected 0 (T_0_), 1 and 3 h after the removal of BafA1.
