# Supplementary figures and images for: Elongation factor 2 kinase promotes cell survival by inhibiting protein synthesis without inducing autophagy
Source: Cell Signal. 2016 Apr;28(4):284–93. doi: 10.1016/j.cellsig.2016.01.005 (PMC4760274; doi:10.1016/j.cellsig.2016.01.005)

Fig. S1

**A**

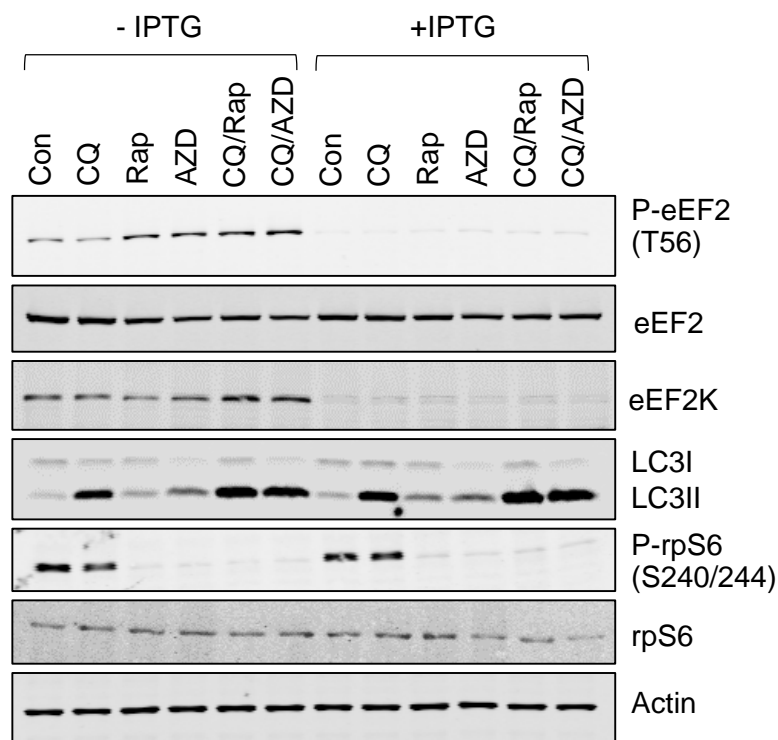

**B**

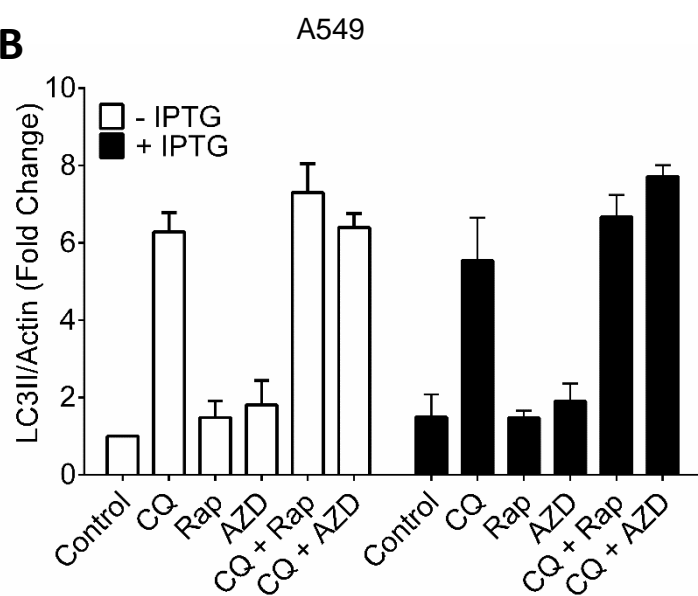

Fig. S2

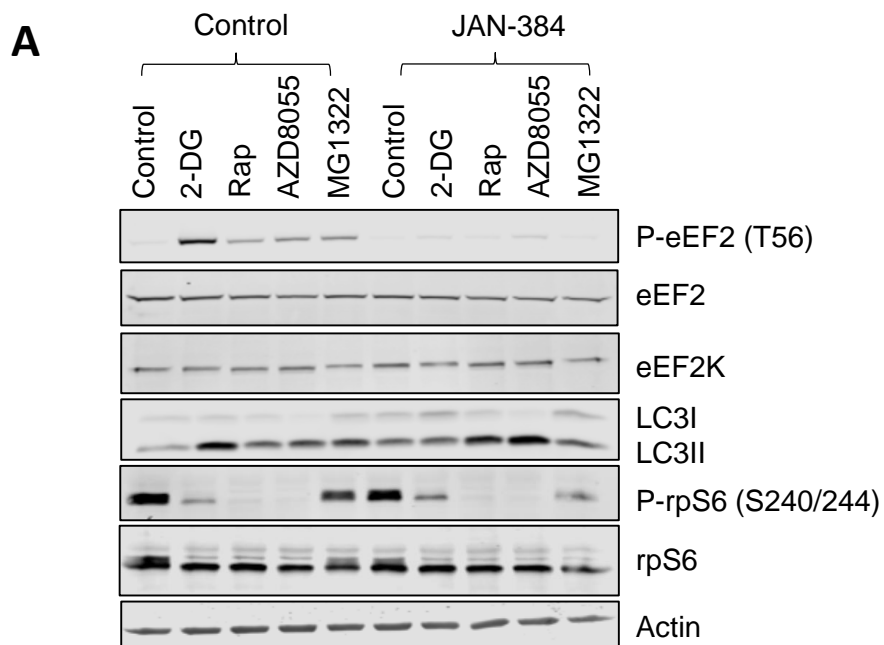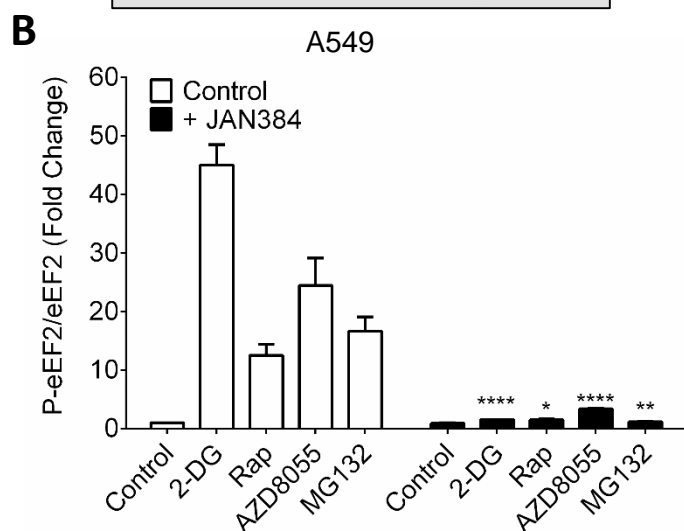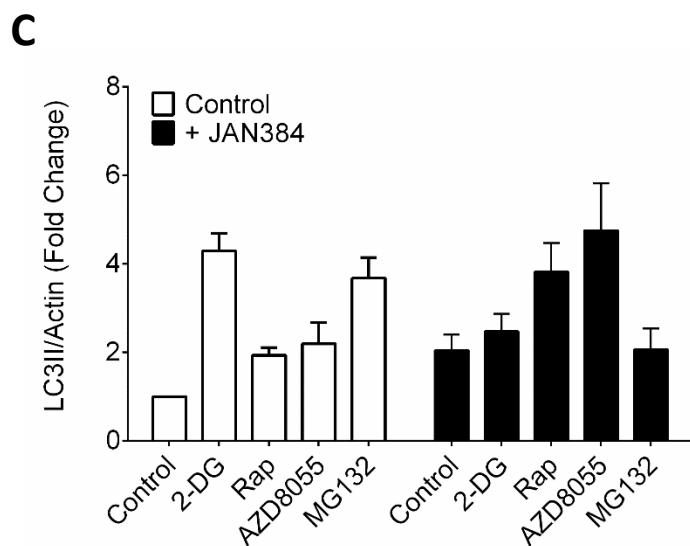

Fig. S3

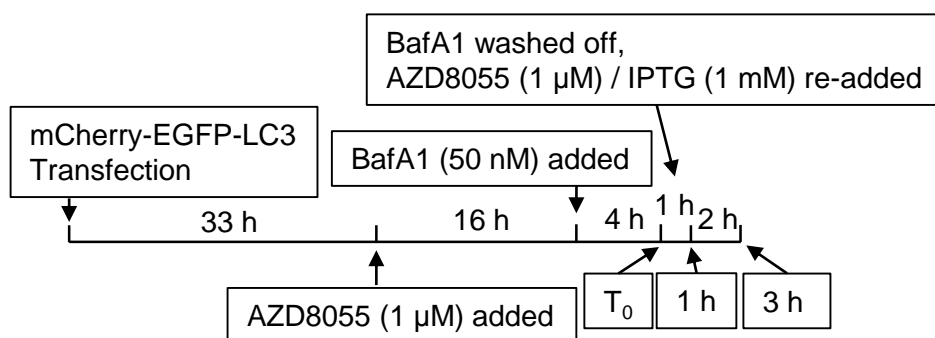

Supplement: Supplementary file 1 — Fig. S1 Role of eEF2K in autophagy in A549 cells (A) A549 cells were cultured in the presence or absence of 1 mM IPTG for 5 days to induce the knockdown of eEF2K. Cells were treated, where indicated, with rapamycin (50 nM) or AZD8055 (0.5 μM) or chloroquine (10 μM) for 16 h. Cells were then lysed and samples containing equal amounts of protein were analysed by western blot using the indicated antibodies. (B) Quantitation of data from multiple experiments as in (C) expressed as LC3II normalized to actin, mean ± SEM (control cells without treatment = 1; n = 3). Fig. S2 Role of eEF2K in autophagy in A549 cells (A) A549 cells were cultured in the presence or absence of 3 μM JAN-384 for 30 minutes prior to treatment with 10 mM 2-deoxyglucose (2DG), rapamycin (100 nM), AZD8055 (1 μM) or MG132 (10 μM) for 16 h. Cells were then lysed and samples containing equal amounts of protein were analysed by western blot using the indicated antibodies. (B) Quantitation of data from multiple experiments as in (A) expressed as P-eEF2 normalized to eEF2, mean ± SEM (control cells without treatment = 1; n = 3). Statistical analysis refers to differences between individual treatments with/without JAN-384, *P < 0.05, **P < 0.01, ****P < 0.0001. (C) Quantitation of data from multiple experiments as in (A) expressed as LC3II normalized to actin, mean ± SEM (control cells without treatment = 1; n = 3). Fig. S3 eEF2K does not play a role in regulating autophagolysosome formation. Schematic representation of the experimental design in Fig. 2C. A549 cells (where indicated, in medium containing 1 mM IPTG to induce the expression of shRNA against eEF2K) were transfected with mCherry-EGFP-LC3B, a tandem fluorescently-tagged LC3. 33 h later cells were treated with vehicle (DMSO) or AZD8055 (1 μM) for 16 h, before the addition of Bafilomycin A1 (BafA1, 50 nM). BafA1 were removed 4 h post-treatment while the vehicle/AZD8055 and IPTG were re-added to the cells. Samples were collected 0 (T0), 1 and 3 h a [file mmc1.pdf]
